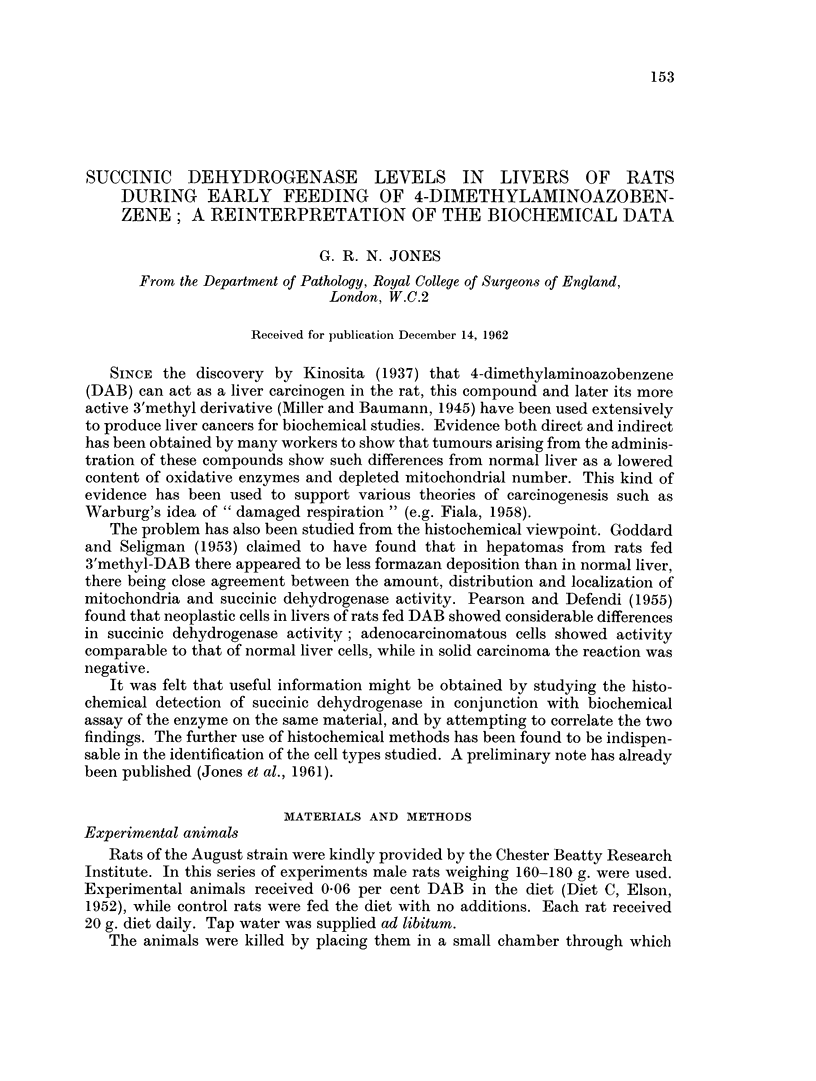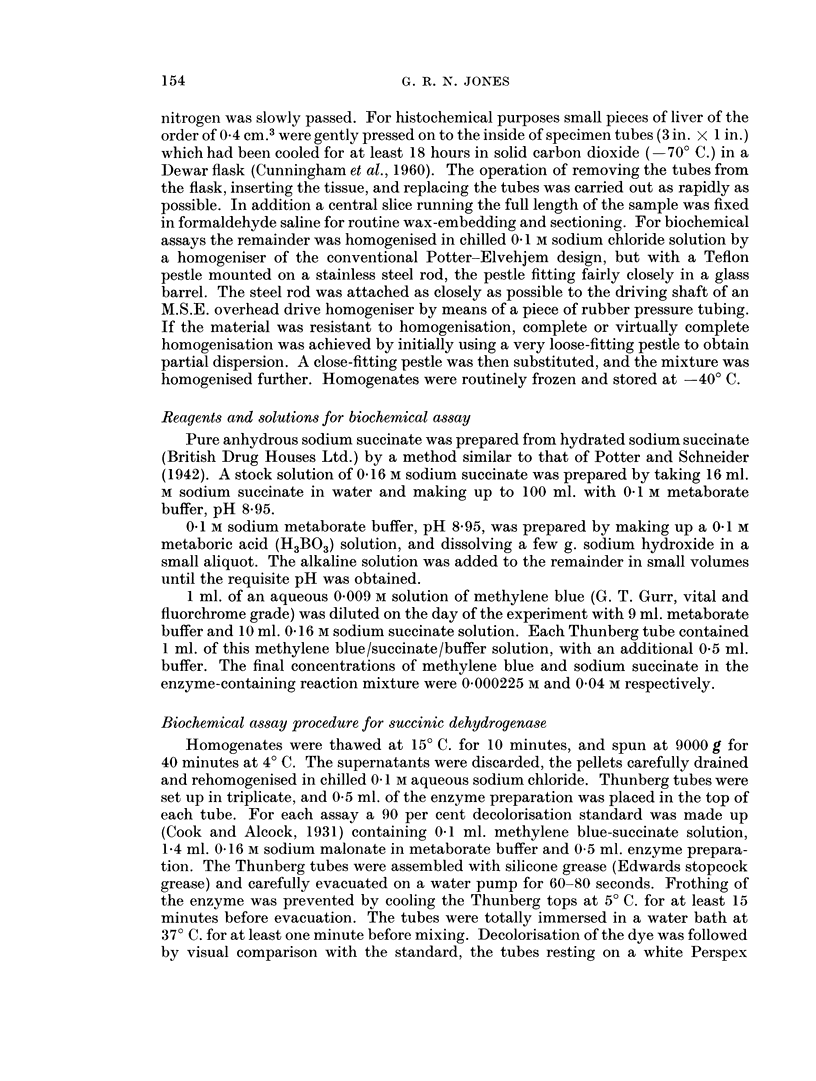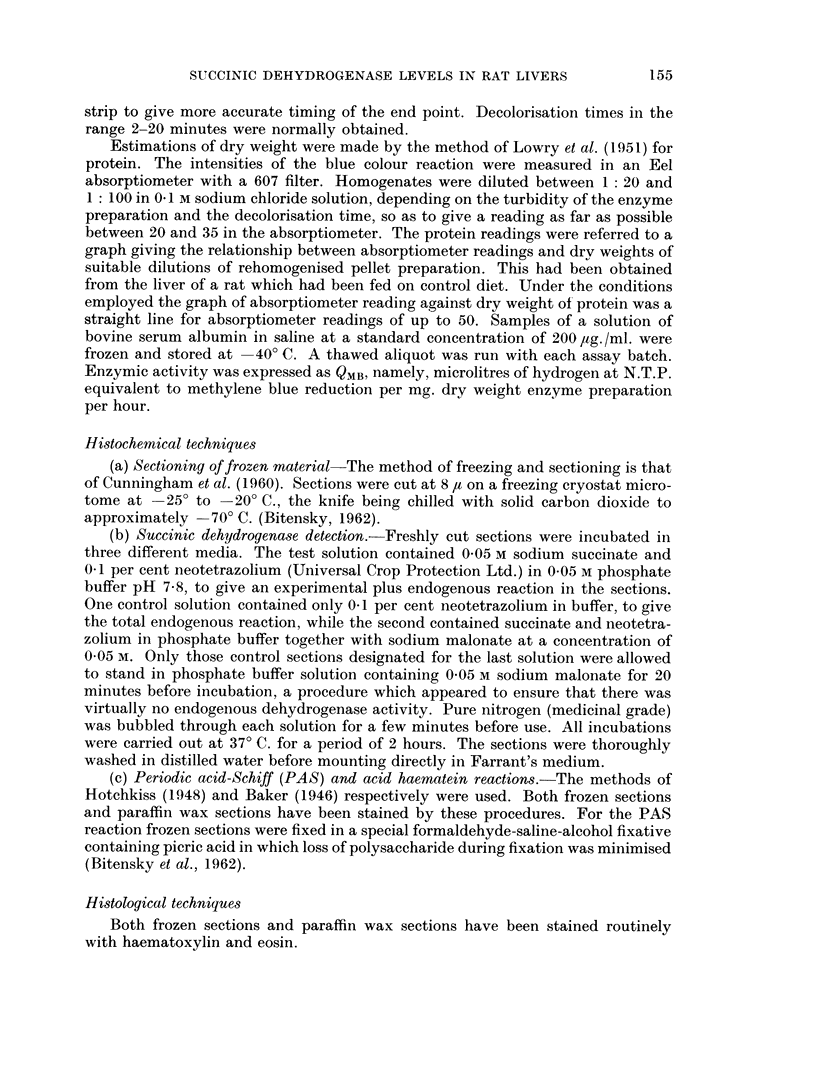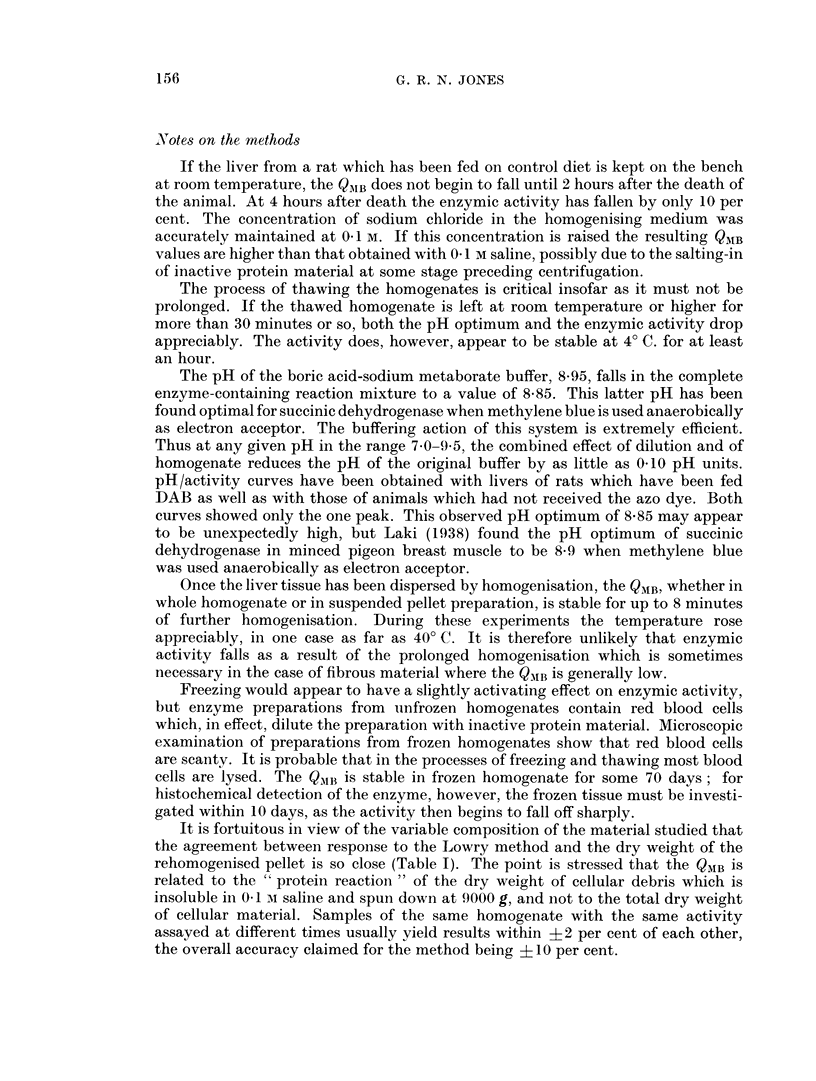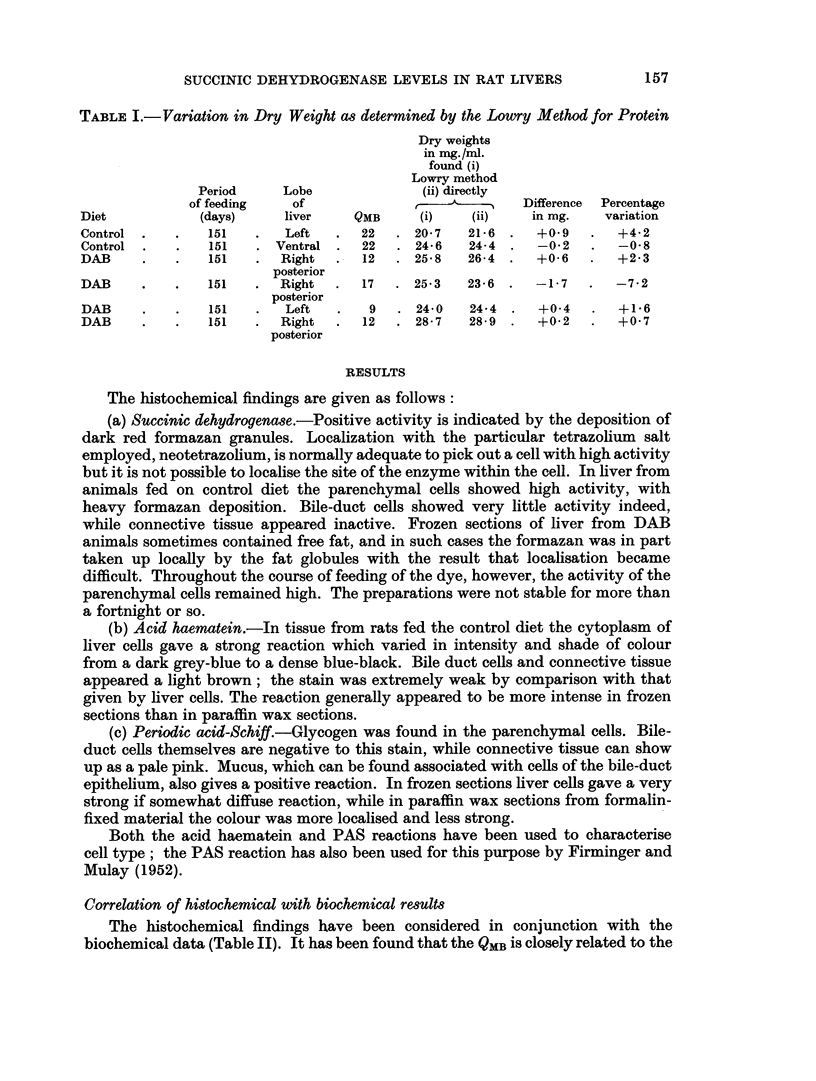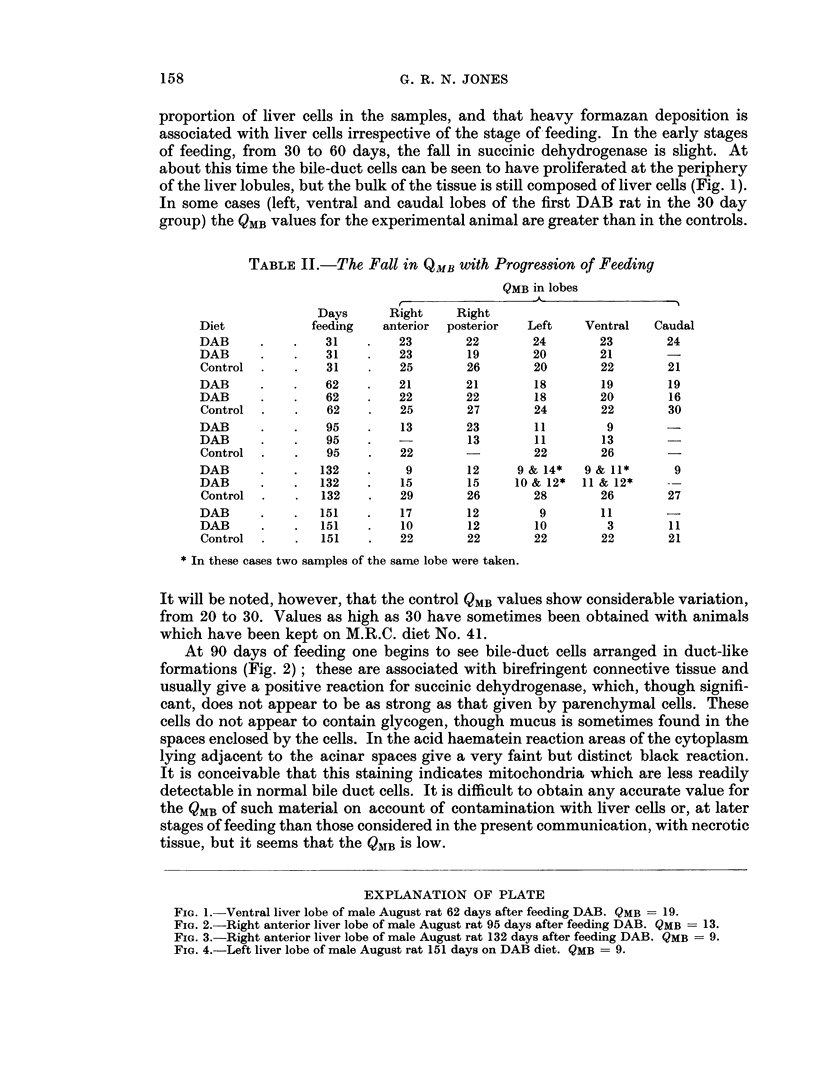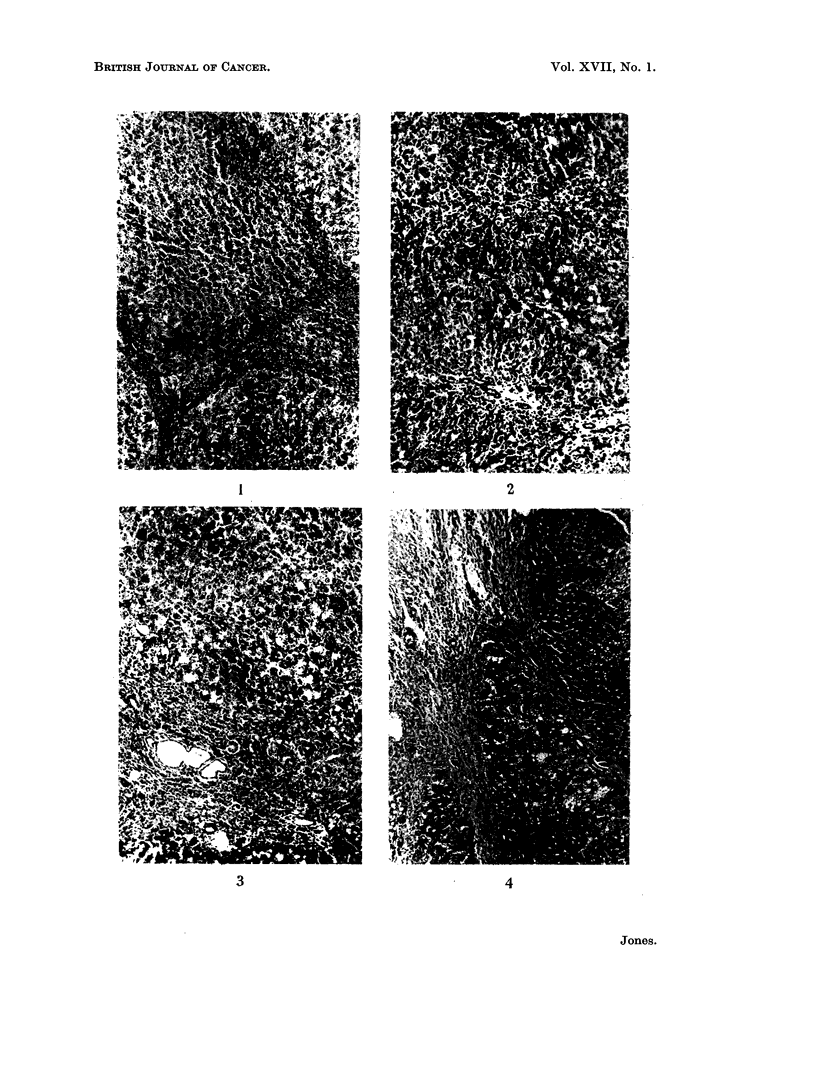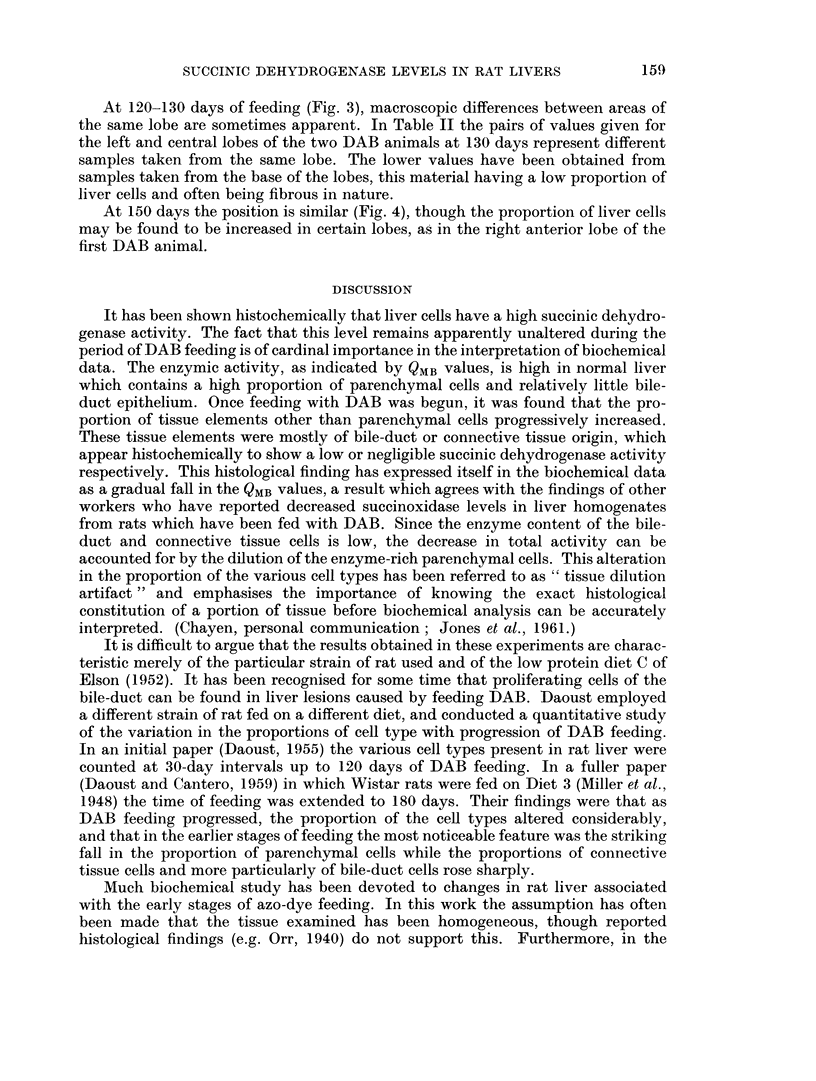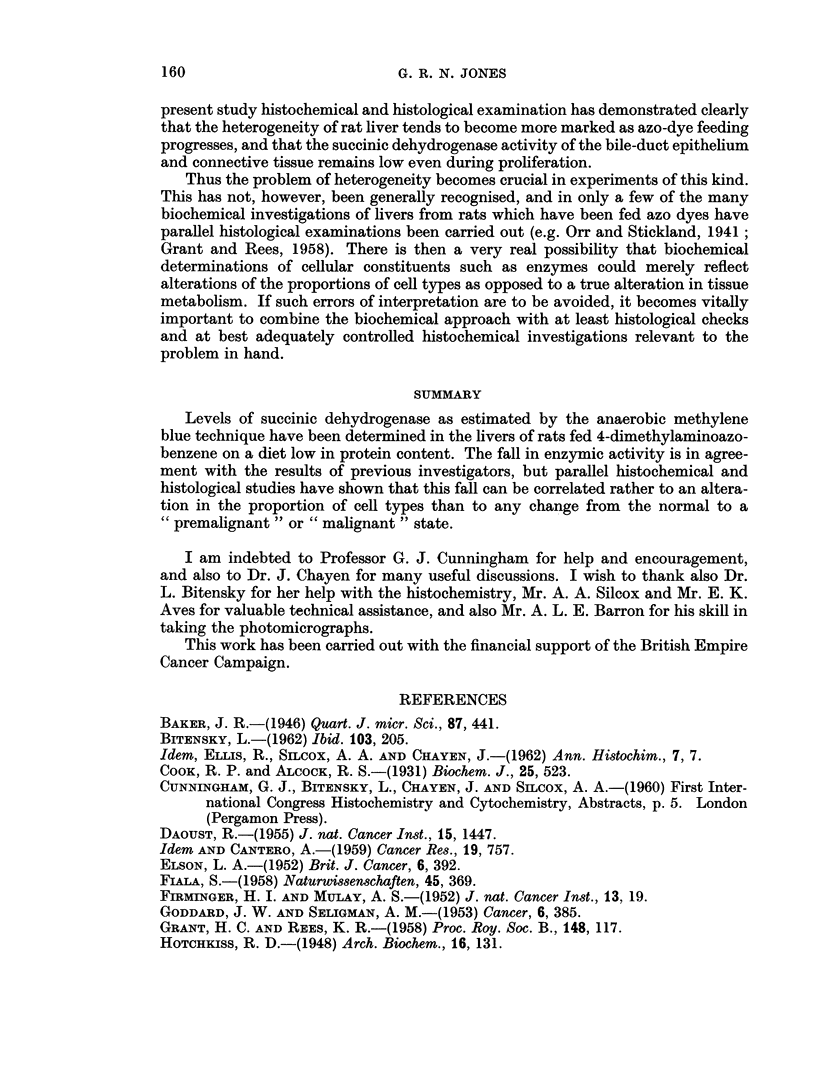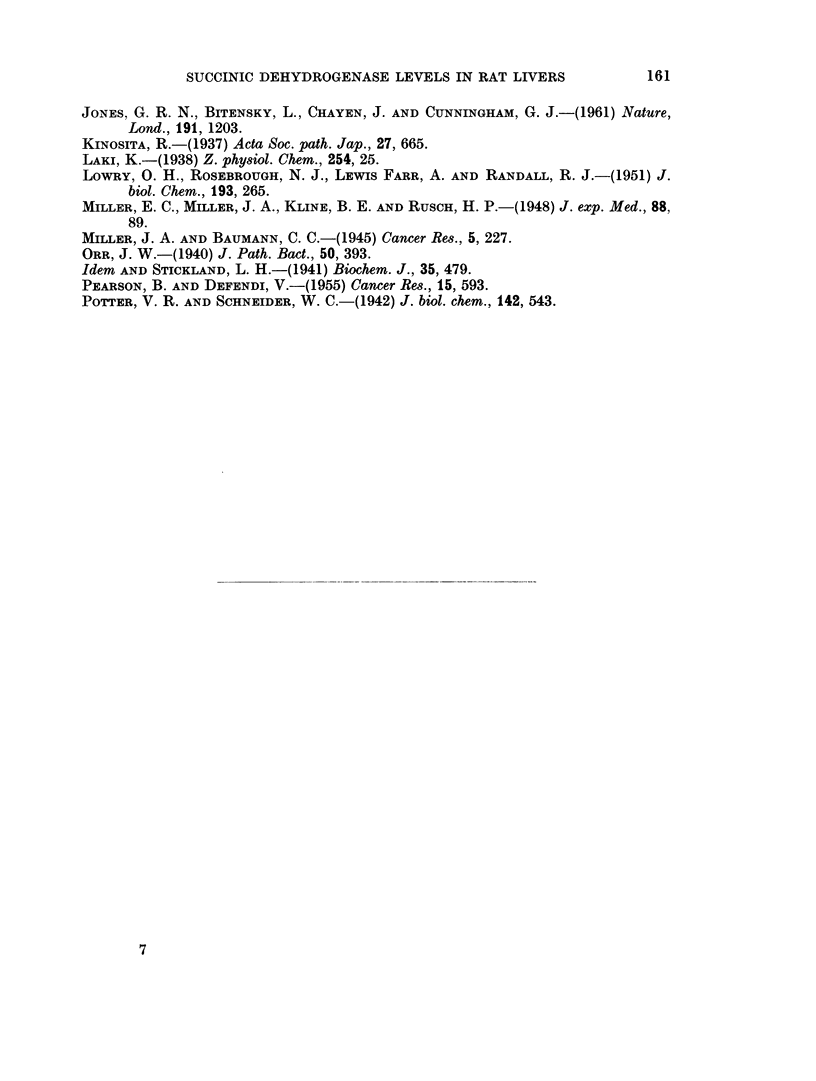# Succinic Dehydrogenase Levels in Livers of Rats During Early Feeding of 4-Dimethylaminoazobenzene; a Reinterpretation of the Biochemical Data

**DOI:** 10.1038/bjc.1963.23

**Published:** 1963-03

**Authors:** G. R. N. Jones

## Abstract

**Images:**


### SUCCINIC DEHYDROGENASE LEVELS IN LIVERS OF RATS DURING EARLY FEEDING OF 4-DIMETHYLAMINOAZOBENZENE ; A REINTERPRETATION OF THE BIOCHEMICAL DATA

G. R. N. JONES

*From the Department of Pathology, Royal College of Surgeons of England, London, W.C.2*

Received for publication December 14, 1962

SINCE the discovery by Kinosita (1937) that 4-dimethylaminoazobenzene (DAB) can act as a liver carcinogen in the rat, this compound and later its more active 3′methyl derivative (Miller and Baumann, 1945) have been used extensively to produce liver cancers for biochemical studies. Evidence both direct and indirect has been obtained by many workers to show that tumours arising from the administration of these compounds show such differences from normal liver as a lowered content of oxidative enzymes and depleted mitochondrial number. This kind of evidence has been used to support various theories of carcinogenesis such as Warburg's idea of " damaged respiration " (e.g. Fiala, 1958).

The problem has also been studied from the histochemical viewpoint. Goddard and Seligman (1953) claimed to have found that in hepatomas from rats fed 3′methyl-DAB there appeared to be less formazan deposition than in normal liver, there being close agreement between the amount, distribution and localization of mitochondria and succinic dehydrogenase activity. Pearson and Defendi (1955) found that neoplastic cells in livers of rats fed DAB showed considerable differences in succinic dehydrogenase activity ; adenocarcinomatous cells showed activity comparable to that of normal liver cells, while in solid carcinoma the reaction was negative.

It was felt that useful information might be obtained by studying the histochemical detection of succinic dehydrogenase in conjunction with biochemical assay of the enzyme on the same material, and by attempting to correlate the two findings. The further use of histochemical methods has been found to be indispensable in the identification of the cell types studied. A preliminary note has already been published (Jones *et al.*, 1961).

### MATERIALS AND METHODS

### *Experimental animals*

Rats of the August strain were kindly provided by the Chester Beatty Research Institute. In this series of experiments male rats weighing 160–180 g. were used. Experimental animals received 0·06 per cent DAB in the diet (Diet C, Elson, 1952), while control rats were fed the diet with no additions. Each rat received 20 g. diet daily. Tap water was supplied *ad libitum.*

The animals were killed by placing them in a small chamber through which nitrogen was slowly passed. For histochemical purposes small pieces of liver of the order of 0·4 $cm.^3$ were gently pressed on to the inside of specimen tubes (3 in. × 1 in.) which had been cooled for at least 18 hours in solid carbon dioxide (−70° C.) in a Dewar flask (Cunningham *et al.*, 1960). The operation of removing the tubes from the flask, inserting the tissue, and replacing the tubes was carried out as rapidly as possible. In addition a central slice running the full length of the sample was fixed in formaldehyde saline for routine wax-embedding and sectioning. For biochemical assays the remainder was homogenised in chilled 0·1 M sodium chloride solution by a homogeniser of the conventional Potter–Elvehjem design, but with a Teflon pestle mounted on a stainless steel rod, the pestle fitting fairly closely in a glass barrel. The steel rod was attached as closely as possible to the driving shaft of an M.S.E. overhead drive homogeniser by means of a piece of rubber pressure tubing. If the material was resistant to homogenisation, complete or virtually complete homogenisation was achieved by initially using a very loose-fitting pestle to obtain partial dispersion. A close-fitting pestle was then substituted, and the mixture was homogenised further. Homogenates were routinely frozen and stored at −40° C.

*Reagents and solutions for biochemical assay*

Pure anhydrous sodium succinate was prepared from hydrated sodium succinate (British Drug Houses Ltd.) by a method similar to that of Potter and Schneider (1942). A stock solution of 0·16 M sodium succinate was prepared by taking 16 ml. M sodium succinate in water and making up to 100 ml. with 0·1 M metaborate buffer, pH 8·95.

0·1 M sodium metaborate buffer, pH 8·95, was prepared by making up a 0·1 M metaboric acid ($H_3BO_3$) solution, and dissolving a few g. sodium hydroxide in a small aliquot. The alkaline solution was added to the remainder in small volumes until the requisite pH was obtained.

1 ml. of an aqueous 0·009 M solution of methylene blue (G. T. Gurr, vital and fluorchrome grade) was diluted on the day of the experiment with 9 ml. metaborate buffer and 10 ml. 0·16 M sodium succinate solution. Each Thunberg tube contained 1 ml. of this methylene blue/succinate/buffer solution, with an additional 0·5 ml. buffer. The final concentrations of methylene blue and sodium succinate in the enzyme-containing reaction mixture were 0·000225 M and 0·04 M respectively.

*Biochemical assay procedure for succinic dehydrogenase*

Homogenates were thawed at 15° C. for 10 minutes, and spun at 9000 ***g*** for 40 minutes at 4° C. The supernatants were discarded, the pellets carefully drained and rehomogenised in chilled 0·1 M aqueous sodium chloride. Thunberg tubes were set up in triplicate, and 0·5 ml. of the enzyme preparation was placed in the top of each tube. For each assay a 90 per cent decolorisation standard was made up (Cook and Alcock, 1931) containing 0·1 ml. methylene blue-succinate solution, 1·4 ml. 0·16 M sodium malonate in metaborate buffer and 0·5 ml. enzyme preparation. The Thunberg tubes were assembled with silicone grease (Edwards stopcock grease) and carefully evacuated on a water pump for 60–80 seconds. Frothing of the enzyme was prevented by cooling the Thunberg tops at 5° C. for at least 15 minutes before evacuation. The tubes were totally immersed in a water bath at 37° C. for at least one minute before mixing. Decolorisation of the dye was followed by visual comparison with the standard, the tubes resting on a white Perspex strip to give more accurate timing of the end point. Decolorisation times in the range 2–20 minutes were normally obtained.

Estimations of dry weight were made by the method of Lowry *et al.* (1951) for protein. The intensities of the blue colour reaction were measured in an Eel absorptiometer with a 607 filter. Homogenates were diluted between 1 : 20 and 1 : 100 in 0·1 M sodium chloride solution, depending on the turbidity of the enzyme preparation and the decolorisation time, so as to give a reading as far as possible between 20 and 35 in the absorptiometer. The protein readings were referred to a graph giving the relationship between absorptiometer readings and dry weights of suitable dilutions of rehomogenised pellet preparation. This had been obtained from the liver of a rat which had been fed on control diet. Under the conditions employed the graph of absorptiometer reading against dry weight of protein was a straight line for absorptiometer readings of up to 50. Samples of a solution of bovine serum albumin in saline at a standard concentration of 200 $\mu$g./ml. were frozen and stored at $-40°$ C. A thawed aliquot was run with each assay batch. Enzymic activity was expressed as $Q_{MB}$, namely, microlitres of hydrogen at N.T.P. equivalent to methylene blue reduction per mg. dry weight enzyme preparation per hour.

*Histochemical techniques*

(a) *Sectioning of frozen material*—The method of freezing and sectioning is that of Cunningham *et al.* (1960). Sections were cut at 8 $\mu$ on a freezing cryostat microtome at $-25°$ to $-20°$ C., the knife being chilled with solid carbon dioxide to approximately $-70°$ C. (Bitensky, 1962).

(b) *Succinic dehydrogenase detection.*—Freshly cut sections were incubated in three different media. The test solution contained 0·05 M sodium succinate and 0·1 per cent neotetrazolium (Universal Crop Protection Ltd.) in 0·05 M phosphate buffer pH 7·8, to give an experimental plus endogenous reaction in the sections. One control solution contained only 0·1 per cent neotetrazolium in buffer, to give the total endogenous reaction, while the second contained succinate and neotetrazolium in phosphate buffer together with sodium malonate at a concentration of 0·05 M. Only those control sections designated for the last solution were allowed to stand in phosphate buffer solution containing 0·05 M sodium malonate for 20 minutes before incubation, a procedure which appeared to ensure that there was virtually no endogenous dehydrogenase activity. Pure nitrogen (medicinal grade) was bubbled through each solution for a few minutes before use. All incubations were carried out at 37° C. for a period of 2 hours. The sections were thoroughly washed in distilled water before mounting directly in Farrant's medium.

(c) *Periodic acid-Schiff (PAS) and acid haematein reactions.*—The methods of Hotchkiss (1948) and Baker (1946) respectively were used. Both frozen sections and paraffin wax sections have been stained by these procedures. For the PAS reaction frozen sections were fixed in a special formaldehyde-saline-alcohol fixative containing picric acid in which loss of polysaccharide during fixation was minimised (Bitensky *et al.*, 1962).

*Histological techniques*

Both frozen sections and paraffin wax sections have been stained routinely with haematoxylin and eosin.

*Notes on the methods*

If the liver from a rat which has been fed on control diet is kept on the bench at room temperature, the $Q_{MB}$ does not begin to fall until 2 hours after the death of the animal. At 4 hours after death the enzymic activity has fallen by only 10 per cent. The concentration of sodium chloride in the homogenising medium was accurately maintained at 0·1 M. If this concentration is raised the resulting $Q_{MB}$ values are higher than that obtained with 0·1 M saline, possibly due to the salting-in of inactive protein material at some stage preceding centrifugation.

The process of thawing the homogenates is critical insofar as it must not be prolonged. If the thawed homogenate is left at room temperature or higher for more than 30 minutes or so, both the pH optimum and the enzymic activity drop appreciably. The activity does, however, appear to be stable at 4° C. for at least an hour.

The pH of the boric acid-sodium metaborate buffer, 8·95, falls in the complete enzyme-containing reaction mixture to a value of 8·85. This latter pH has been found optimal for succinic dehydrogenase when methylene blue is used anaerobically as electron acceptor. The buffering action of this system is extremely efficient. Thus at any given pH in the range 7·0–9·5, the combined effect of dilution and of homogenate reduces the pH of the original buffer by as little as 0·10 pH units. pH/activity curves have been obtained with livers of rats which have been fed DAB as well as with those of animals which had not received the azo dye. Both curves showed only the one peak. This observed pH optimum of 8·85 may appear to be unexpectedly high, but Laki (1938) found the pH optimum of succinic dehydrogenase in minced pigeon breast muscle to be 8·9 when methylene blue was used anaerobically as electron acceptor.

Once the liver tissue has been dispersed by homogenisation, the $Q_{MB}$, whether in whole homogenate or in suspended pellet preparation, is stable for up to 8 minutes of further homogenisation. During these experiments the temperature rose appreciably, in one case as far as 40° C. It is therefore unlikely that enzymic activity falls as a result of the prolonged homogenisation which is sometimes necessary in the case of fibrous material where the $Q_{MB}$ is generally low.

Freezing would appear to have a slightly activating effect on enzymic activity, but enzyme preparations from unfrozen homogenates contain red blood cells which, in effect, dilute the preparation with inactive protein material. Microscopic examination of preparations from frozen homogenates show that red blood cells are scanty. It is probable that in the processes of freezing and thawing most blood cells are lysed. The $Q_{MB}$ is stable in frozen homogenate for some 70 days ; for histochemical detection of the enzyme, however, the frozen tissue must be investigated within 10 days, as the activity then begins to fall off sharply.

It is fortuitous in view of the variable composition of the material studied that the agreement between response to the Lowry method and the dry weight of the rehomogenised pellet is so close (Table I). The point is stressed that the $Q_{MB}$ is related to the " protein reaction " of the dry weight of cellular debris which is insoluble in 0·1 M saline and spun down at 9000 *g*, and not to the total dry weight of cellular material. Samples of the same homogenate with the same activity assayed at different times usually yield results within $\pm 2$ per cent of each other, the overall accuracy claimed for the method being $\pm 10$ per cent.

TABLE I.—*Variation in Dry Weight as determined by the Lowry Method for Protein*

| Diet | Period of feeding (days) | Lobe of liver | $Q_{MB}$ | Dry weights in mg./ml. found (i) Lowry method (ii) directly (i) | (ii) | Difference in mg. | Percentage variation |
|---|---|---|---|---|---|---|---|
| Control | 151 | Left | 22 | 20·7 | 21·6 | +0·9 | +4·2 |
| Control | 151 | Ventral | 22 | 24·6 | 24·4 | −0·2 | −0·8 |
| DAB | 151 | Right posterior | 12 | 25·8 | 26·4 | +0·6 | +2·3 |
| DAB | 151 | Right posterior | 17 | 25·3 | 23·6 | −1·7 | −7·2 |
| DAB | 151 | Left | 9 | 24·0 | 24·4 | +0·4 | +1·6 |
| DAB | 151 | Right posterior | 12 | 28·7 | 28·9 | +0·2 | +0·7 |

### RESULTS

The histochemical findings are given as follows :

(a) *Succinic dehydrogenase.*—Positive activity is indicated by the deposition of dark red formazan granules. Localization with the particular tetrazolium salt employed, neotetrazolium, is normally adequate to pick out a cell with high activity but it is not possible to localise the site of the enzyme within the cell. In liver from animals fed on control diet the parenchymal cells showed high activity, with heavy formazan deposition. Bile-duct cells showed very little activity indeed, while connective tissue appeared inactive. Frozen sections of liver from DAB animals sometimes contained free fat, and in such cases the formazan was in part taken up locally by the fat globules with the result that localisation became difficult. Throughout the course of feeding of the dye, however, the activity of the parenchymal cells remained high. The preparations were not stable for more than a fortnight or so.

(b) *Acid haematein.*—In tissue from rats fed the control diet the cytoplasm of liver cells gave a strong reaction which varied in intensity and shade of colour from a dark grey-blue to a dense blue-black. Bile duct cells and connective tissue appeared a light brown ; the stain was extremely weak by comparison with that given by liver cells. The reaction generally appeared to be more intense in frozen sections than in paraffin wax sections.

(c) *Periodic acid-Schiff.*—Glycogen was found in the parenchymal cells. Bile-duct cells themselves are negative to this stain, while connective tissue can show up as a pale pink. Mucus, which can be found associated with cells of the bile-duct epithelium, also gives a positive reaction. In frozen sections liver cells gave a very strong if somewhat diffuse reaction, while in paraffin wax sections from formalin-fixed material the colour was more localised and less strong.

Both the acid haematein and PAS reactions have been used to characterise cell type ; the PAS reaction has also been used for this purpose by Firminger and Mulay (1952).

### *Correlation of histochemical with biochemical results*

The histochemical findings have been considered in conjunction with the biochemical data (Table II). It has been found that the $Q_{MB}$ is closely related to the proportion of liver cells in the samples, and that heavy formazan deposition is associated with liver cells irrespective of the stage of feeding. In the early stages of feeding, from 30 to 60 days, the fall in succinic dehydrogenase is slight. At about this time the bile-duct cells can be seen to have proliferated at the periphery of the liver lobules, but the bulk of the tissue is still composed of liver cells (Fig. 1). In some cases (left, ventral and caudal lobes of the first DAB rat in the 30 day group) the $Q_{MB}$ values for the experimental animal are greater than in the controls.

TABLE II.—*The Fall in* $Q_{MB}$ *with Progression of Feeding*

| Diet | Days feeding | $Q_{MB}$ in lobes: Right anterior | Right posterior | Left | Ventral | Caudal |
|---|---|---|---|---|---|---|
| DAB | 31 | 23 | 22 | 24 | 23 | 24 |
| DAB | 31 | 23 | 19 | 20 | 21 | — |
| Control | 31 | 25 | 26 | 20 | 22 | 21 |
| DAB | 62 | 21 | 21 | 18 | 19 | 19 |
| DAB | 62 | 22 | 22 | 18 | 20 | 16 |
| Control | 62 | 25 | 27 | 24 | 22 | 30 |
| DAB | 95 | 13 | 23 | 11 | 9 | — |
| DAB | 95 | — | 13 | 11 | 13 | — |
| Control | 95 | 22 | — | 22 | 26 | — |
| DAB | 132 | 9 | 12 | 9 & 14* | 9 & 11* | 9 |
| DAB | 132 | 15 | 15 | 10 & 12* | 11 & 12* | — |
| Control | 132 | 29 | 26 | 28 | 26 | 27 |
| DAB | 151 | 17 | 12 | 9 | 11 | — |
| DAB | 151 | 10 | 12 | 10 | 3 | 11 |
| Control | 151 | 22 | 22 | 22 | 22 | 21 |

* In these cases two samples of the same lobe were taken.

It will be noted, however, that the control $Q_{MB}$ values show considerable variation, from 20 to 30. Values as high as 30 have sometimes been obtained with animals which have been kept on M.R.C. diet No. 41.

At 90 days of feeding one begins to see bile-duct cells arranged in duct-like formations (Fig. 2); these are associated with birefringent connective tissue and usually give a positive reaction for succinic dehydrogenase, which, though significant, does not appear to be as strong as that given by parenchymal cells. These cells do not appear to contain glycogen, though mucus is sometimes found in the spaces enclosed by the cells. In the acid haematein reaction areas of the cytoplasm lying adjacent to the acinar spaces give a very faint but distinct black reaction. It is conceivable that this staining indicates mitochondria which are less readily detectable in normal bile duct cells. It is difficult to obtain any accurate value for the $Q_{MB}$ of such material on account of contamination with liver cells or, at later stages of feeding than those considered in the present communication, with necrotic tissue, but it seems that the $Q_{MB}$ is low.

---

EXPLANATION OF PLATE

FIG. 1.—Ventral liver lobe of male August rat 62 days after feeding DAB. $Q_{MB} = 19$.
FIG. 2.—Right anterior liver lobe of male August rat 95 days after feeding DAB. $Q_{MB} = 13$.
FIG. 3.—Right anterior liver lobe of male August rat 132 days after feeding DAB. $Q_{MB} = 9$.
FIG. 4.—Left liver lobe of male August rat 151 days on DAB diet. $Q_{MB} = 9$.

1

2

3

4

At 120–130 days of feeding (Fig. 3), macroscopic differences between areas of the same lobe are sometimes apparent. In Table II the pairs of values given for the left and central lobes of the two DAB animals at 130 days represent different samples taken from the same lobe. The lower values have been obtained from samples taken from the base of the lobes, this material having a low proportion of liver cells and often being fibrous in nature.

At 150 days the position is similar (Fig. 4), though the proportion of liver cells may be found to be increased in certain lobes, as in the right anterior lobe of the first DAB animal.

### DISCUSSION

It has been shown histochemically that liver cells have a high succinic dehydrogenase activity. The fact that this level remains apparently unaltered during the period of DAB feeding is of cardinal importance in the interpretation of biochemical data. The enzymic activity, as indicated by $Q_{MB}$ values, is high in normal liver which contains a high proportion of parenchymal cells and relatively little bile-duct epithelium. Once feeding with DAB was begun, it was found that the proportion of tissue elements other than parenchymal cells progressively increased. These tissue elements were mostly of bile-duct or connective tissue origin, which appear histochemically to show a low or negligible succinic dehydrogenase activity respectively. This histological finding has expressed itself in the biochemical data as a gradual fall in the $Q_{MB}$ values, a result which agrees with the findings of other workers who have reported decreased succinoxidase levels in liver homogenates from rats which have been fed with DAB. Since the enzyme content of the bile-duct and connective tissue cells is low, the decrease in total activity can be accounted for by the dilution of the enzyme-rich parenchymal cells. This alteration in the proportion of the various cell types has been referred to as " tissue dilution artifact " and emphasises the importance of knowing the exact histological constitution of a portion of tissue before biochemical analysis can be accurately interpreted. (Chayen, personal communication ; Jones *et al.*, 1961.)

It is difficult to argue that the results obtained in these experiments are characteristic merely of the particular strain of rat used and of the low protein diet C of Elson (1952). It has been recognised for some time that proliferating cells of the bile-duct can be found in liver lesions caused by feeding DAB. Daoust employed a different strain of rat fed on a different diet, and conducted a quantitative study of the variation in the proportions of cell type with progression of DAB feeding. In an initial paper (Daoust, 1955) the various cell types present in rat liver were counted at 30-day intervals up to 120 days of DAB feeding. In a fuller paper (Daoust and Cantero, 1959) in which Wistar rats were fed on Diet 3 (Miller *et al.*, 1948) the time of feeding was extended to 180 days. Their findings were that as DAB feeding progressed, the proportion of the cell types altered considerably, and that in the earlier stages of feeding the most noticeable feature was the striking fall in the proportion of parenchymal cells while the proportions of connective tissue cells and more particularly of bile-duct cells rose sharply.

Much biochemical study has been devoted to changes in rat liver associated with the early stages of azo-dye feeding. In this work the assumption has often been made that the tissue examined has been homogeneous, though reported histological findings (e.g. Orr, 1940) do not support this. Furthermore, in the present study histochemical and histological examination has demonstrated clearly that the heterogeneity of rat liver tends to become more marked as azo-dye feeding progresses, and that the succinic dehydrogenase activity of the bile-duct epithelium and connective tissue remains low even during proliferation.

Thus the problem of heterogeneity becomes crucial in experiments of this kind. This has not, however, been generally recognised, and in only a few of the many biochemical investigations of livers from rats which have been fed azo dyes have parallel histological examinations been carried out (e.g. Orr and Stickland, 1941; Grant and Rees, 1958). There is then a very real possibility that biochemical determinations of cellular constituents such as enzymes could merely reflect alterations of the proportions of cell types as opposed to a true alteration in tissue metabolism. If such errors of interpretation are to be avoided, it becomes vitally important to combine the biochemical approach with at least histological checks and at best adequately controlled histochemical investigations relevant to the problem in hand.

### SUMMARY

Levels of succinic dehydrogenase as estimated by the anaerobic methylene blue technique have been determined in the livers of rats fed 4-dimethylaminoazobenzene on a diet low in protein content. The fall in enzymic activity is in agreement with the results of previous investigators, but parallel histochemical and histological studies have shown that this fall can be correlated rather to an alteration in the proportion of cell types than to any change from the normal to a "premalignant" or "malignant" state.

I am indebted to Professor G. J. Cunningham for help and encouragement, and also to Dr. J. Chayen for many useful discussions. I wish to thank also Dr. L. Bitensky for her help with the histochemistry, Mr. A. A. Silcox and Mr. E. K. Aves for valuable technical assistance, and also Mr. A. L. E. Barron for his skill in taking the photomicrographs.

This work has been carried out with the financial support of the British Empire Cancer Campaign.

### REFERENCES

BAKER, J. R.—(1946) *Quart. J. micr. Sci.*, **87**, 441.
BITENSKY, L.—(1962) *Ibid.* **103**, 205.
*Idem*, ELLIS, R., SILCOX, A. A. AND CHAYEN, J.—(1962) *Ann. Histochim.*, **7**, 7.
COOK, R. P. and ALCOCK, R. S.—(1931) *Biochem. J.*, **25**, 523.
CUNNINGHAM, G. J., BITENSKY, L., CHAYEN, J. AND SILCOX, A. A.—(1960) First International Congress Histochemistry and Cytochemistry, Abstracts, p. 5. London (Pergamon Press).
DAOUST, R.—(1955) *J. nat. Cancer Inst.*, **15**, 1447.
*Idem* AND CANTERO, A.—(1959) *Cancer Res.*, **19**, 757.
ELSON, L. A.—(1952) *Brit. J. Cancer*, **6**, 392.
FIALA, S.—(1958) *Naturwissenschaften*, **45**, 369.
FIRMINGER, H. I. AND MULAY, A. S.—(1952) *J. nat. Cancer Inst.*, **13**, 19.
GODDARD, J. W. AND SELIGMAN, A. M.—(1953) *Cancer*, **6**, 385.
GRANT, H. C. AND REES, K. R.—(1958) *Proc. Roy. Soc.* B., **148**, 117.
HOTCHKISS, R. D.—(1948) *Arch. Biochem.*, **16**, 131.

JONES, G. R. N., BITENSKY, L., CHAYEN, J. AND CUNNINGHAM, G. J.—(1961) *Nature, Lond.*, **191**, 1203.
KINOSITA, R.—(1937) *Acta Soc. path. Jap.*, **27**, 665.
LAKI, K.—(1938) *Z. physiol. Chem.*, **254**, 25.
LOWRY, O. H., ROSEBROUGH, N. J., LEWIS FARR, A. AND RANDALL, R. J.—(1951) *J. biol. Chem.*, **193**, 265.
MILLER, E. C., MILLER, J. A., KLINE, B. E. AND RUSCH, H. P.—(1948) *J. exp. Med.*, **88**, 89.
MILLER, J. A. AND BAUMANN, C. C.—(1945) *Cancer Res.*, **5**, 227.
ORR, J. W.—(1940) *J. Path. Bact.*, **50**, 393.
*Idem* AND STICKLAND, L. H.—(1941) *Biochem. J.*, **35**, 479.
PEARSON, B. AND DEFENDI, V.—(1955) *Cancer Res.*, **15**, 593.
POTTER, V. R. AND SCHNEIDER, W. C.—(1942) *J. biol. chem.*, **142**, 543.